# Supplementary material for: Tracking life and death of carbon nitride supports in platinum-catalyzed vinyl chloride synthesis
Source: Nat Commun. 2025 May 24;16:4842. doi: 10.1038/s41467-025-60169-7 (PMC12103515; doi:10.1038/s41467-025-60169-7)
Supplement: Supplementary file 1 — Supplementary information [file 41467_2025_60169_MOESM1_ESM.pdf]

Supplementary Information

Tracking life and death of carbon nitride supports in platinum-catalyzed vinyl chloride synthesis

*Vera Giulimondi,[1,7] Mikhail Agrachev,[2,7] Sergei Kuzin,[2] José Manuel González-Acosta,[3,4] Andrea Ruiz-Ferrando,[3] Frank Krumeich,[5] Federica Bondino,[6] Yung-Tai Chiang,[1,7] Matteo Vanni,[1] Gunnar Jeschke,[2,7] Núria López,[3] and Javier Pérez-Ramírez[1,7]**

[1] Institute for Chemical and Bioengineering, Department of Chemistry and Applied Biosciences, ETH Zürich, Vladimir-Prelog-Weg 1, 8093 Zürich, Switzerland.

[2] Institute of Molecular Physical Science, Department of Chemistry and Applied Biosciences, ETH Zürich, Vladimir-Prelog-Weg 1, 8093 Zürich, Switzerland.

[3] Institute of Chemical Research of Catalonia (ICIQ-CERCA), Av. Països Catalans 16, 43007 Tarragona, Spain.

[4] Department of Physical and Inorganic Chemistry, Universitat Rovira i Virgili, Marcel·lí Domingo s/n, 43007 Tarragona, Spain

[5] Laboratory of Inorganic Chemistry, Department of Chemistry and Applied Biosciences, ETH Zürich, Vladimir-Prelog-Weg 1, 8093 Zürich, Switzerland.

[6] Consiglio Nazionale delle Ricerche (CNR), Istituto Officina dei Materiali (IOM), Strada Statale 14 km 163.5, 34149 Basovizza, Italy.

[7] NCCR Catalysis, Switzerland.

* Corresponding author: jpr@chem.ethz.ch.

Table of contents

1. Supplementary experimental procedures 2

1.1. Catalyst preparation 2

1.2. Catalyst characterization 2

1.3. Catalyst evaluation 5

1.4. Evaluation of mass and heat transfer limitations 6

1.5. Computational methods 7

2. Supplementary tables 9

3. Supplementary figures 20

4. Supplementary references 51

1. Supplementary experimental procedures

1.1. Catalyst preparation

Linear melem oligomers (LMO), partially-polymerized carbon nitride (ppCN) and polymeric carbon nitride (CN) were prepared by calcining melamine (8 g) at 723, 773 and 823 K (heating rate = 2.3 K min^{-1}, dwell time =4 h), respectively, under a nitrogen flow (15 cm^3 min^{-1}). Exfoliated LMO, ppCN, and CN (ELMO, EppCN, ECN) were obtained *via* thermal exfoliation of the powdered bulk materials (<0.3 mm) at 773 K (heating rate = 5 K min^{-1}, hold time = 5 h) in static air. The platinum single-atom catalysts (Pt SACs) were prepared *via* an incipient wetness impregnation method with a nominal metal content of 1 wt%. When indicated, the nominal metal content was varied to maintain a constant metal-content-to-surface-area ratio (*i.e.*, metal density) of 75 μmol_{Pt} m^{-2}. The metal precursor, H_2PtCl_6 (ABCR, 99.9%, 40 wt% Pt), was dissolved in water (1.5 cm^3 g^{-1}) and the obtained solutions were added dropwise, under magnetic stirring, to the carbon nitride supports. Subsequently, all samples were dried at 333 K for 12 h. The respective single-atom catalysts were obtained *via* thermal activation (T_a = 473 K, heating rate = 5 K min^{-1}, hold time = 12 h) in static air and denoted as Pt_{SA}/support (support = LMO, ppCN, CN, ELMO, EppCN, or ECN). Furthermore, a nanoparticle-based catalyst supported on ECN was obtained *via* thermal activation (T_a = 473 K, heating rate = 5 K min^{-1}, hold time = 12 h) in H_2/He (50/50 vol%) and denoted as Pt_{NP}/ECN.

1.2. Catalyst characterization

Nitrogen sorption isotherms were measured at 77 K in a Micromeritics Tristar II, after degassing the solids at 473 K for 12 h.

Powder X-ray diffraction (XRD) was measured using a PANalytical X'Pert PRO-MPD diffractometer with Cu-Kα radiation (λ = 1.54060 Å). The data was recorded in the 10-70° 2θ range with an angular step size of 0.017° and a counting time of 0.26 s per step.

Scanning transmission electron micrographs (STEM) with a high-angle annular dark-field (HAADF) detector were acquired on an aberration-corrected JEOL JEM-ARM300F (GrandARM) microscope operated at 300 kV. Samples were prepared by dipping the copper grid supporting a holey carbon foil in a suspension of the solid in ethanol and drying in air. The nearest-neighbor distributions (NND) between metal centers and their mean value (<NND) were evaluated by analyzing the acquired HAADF-STEM images by an atom detection inference pipeline, accessible through the web app https://huggingface.co/spaces/nccr-catalysis/atom-detection.[1]

The ^{13}C solid-state cross-polarization/magic angle spinning nuclear magnetic resonance (CP/MAS NMR) spectra were recorded on a Bruker AVANCE III HD NMR spectrometer at a magnetic field of 16.4 T corresponding to a ^1H Larmor frequency of 700.13 MHz. A 4-mm double resonance probe head at a spinning speed of 10 kHz was used for all experiments. The ^{13}C spectra were acquired using a cross-polarization experiment with a contact time of 2 ms and a recycle delay of 1 s. A total of 64×103 scans were added for each sample. Between 39×103 and 96×103 scans were acquired, depending on the

sample. The ^{13}C experiments used high-power ^{1}H decoupling during acquisition using a SPINAL-64 sequence.

Fourier transform infrared spectroscopy (FTIR) measurements were conducted with a Bruker Optics Vertex 70 spectrometer equipped with a liquid N_2-cooled mercury cadmium telluride (MCT) detector. Each sample (10 mg) was diluted with KBr (sample:KBr = 1:100 wt/wt), pressed into a self-supporting wafer (area = 1.3 cm^2) using a Specac hydraulic press, applying pressure (3832 bar). The wafers were installed inside a home-built cell equipped with KBr windows and degassed at 3-10 mbar and 473 K, for 2 h. The FTIR spectrum was recorded by co-adding 64 scans in the range of 400-4000 cm^{-1} with a nominal spectral resolution of 4 cm^{-1}.

Ultraviolet-visible diffuse reflectance spectroscopy (UV-vis DRS) measurements of undiluted powder samples were acquired using a JASCO V-770 spectrophotometer equipped with an ILN-725 integrating sphere. The spectra were recorded in the range of 220-800 nm with a data internal of 0.5 nm.

Raman spectroscopy was carried out in a confocal Raman microscope (LabRAM HR Evolution UV-VIS-NIR) using a 325 nm HeCd laser. The microscope was operated in backscattering mode with a 40× objective lens and 0.5 mW power.

X-ray photoelectron spectroscopy (XPS) measurements were conducted on a Physical Electronics Quantum 2000 instrument using monochromatic Al-Kα radiation, generated from an electron beam operated at 15 kV, and equipped with a hemispherical capacitor electron-energy analyzer. The samples were analyzed at an electron take-off angle of 45° and a constant analyzer pass energy of 46.95 eV with a spectra resolution width of 0.2 eV. The XPS spectra were calibrated using the C 1s photoemission, set at 288.3 eV.[2] The Pt 4*f* spectra were fitted after Shirley background subtraction. The selected peak positions are based on literature data.[3]

X-ray absorption spectroscopy (XAS) measurements were performed at the Swiss-Norwegian beamlines (SNBL, BM31) at the European Synchrotron Radiation Facility (ESRF).[4] The X-ray beam was collimated using a double-crystal liquid nitrogen-cooled Si(111) monochromator and calibrated using Pt foil.[4] The gas filling inside the ionization chambers ensured optimal absorption at the Pt L_3 absorption edge. All spectra were recorded in transmission mode at room temperature (ca. 298 K), using a one-element silicon drift detector with Peltier cooling. Continuous scanning was performed for the Pt L_3 edge (between 11.45 and 12.10 keV), and the step size was set to 0.6 eV, with a scan duration of 180 s. The incident X-ray beam was focused on a 3 mm (horizontal) by 0.2 mm (vertical) area. The resulting spectra were energy calibrated, background corrected, normalized, and analyzed using the Demeter software package.[5] k^3-weighted extended X-ray absorption fine structure (EXAFS) spectra were fitted in the optimal k-space (3-10.5 Å^{-1}) and R-space (1.1-3 Å) windows. An amplitude reduction factor of 0.79 was determined by fitting the EXAFS spectrum of a Pt foil.

Soft XAS measurements were performed at the CNR-IOM beamline BACH at the Elettra Synchrotron radiation facility. The energy scale was calibrated to the Au 4*f* 7/2 peak (84.0 eV) measured

on a clean gold reference in electrical contact with the sample. The spectra were recorded in total electron yield, in a normal emission geometry, with the light linearly polarized in the horizontal plane.

Continuous wave (CW) electron paramagnetic resonance (EPR) spectroscopy experiments were performed at room temperature (ca. 298 K) and 10 K on a Bruker Elexsys E500 spectrometer equipped with an Oxford helium (ESR900) cryostat operating at X-band frequencies (≈ 9.4 GHz) using a ER4122SHQE Bruker EPR Resonator. All CW-EPR spectra were acquired (spectrometer settings: microwave frequency = 9.4 GHz, center field = 335.3 mT, sweep width = 200 mT, modulation frequency = 100 kHz, modulation amplitude = 1 G, microwave power = 20.07 mW, 2.007 mW, and 20.07 μW, power attenuation = 10, 20, and 40 dB, conversion time = 40.96 ms, time constant = 20.48 ms), while all measured *g* factors were offset-corrected against a known standard (*i.e.*, free radical 1,1-diphenyl-2-picrylhydrazyl). The EPR spectra were simulated on Matlab using the Easyspin software.[6] The positions of spectral features in EPR spectra are defined by the principal values of the *g*-tensor: a single *g* factor for the isotropic case, g_{xx}, g_{yy} and g_{zz} for the orthorhombic case, g_{II} and $g_{\perp}$ for the axial case. The microwave attenuation has been varied to selectively partially saturate specific spectral components and thus improve the reliability of the fits. Provided the corrosiveness of HCl, an experimental set-up, described elsewhere,[7] was designed and employed for conducting CW-EPR measurements in *operando* mode while ensuring the equipment and personnel safety. For this latter purpose, the gases C_2H_2 (PanGas, purity 2.6), HCl (Air Liquide, 1 vol% in He, anhydrous), Ar (PanGas, purity 5.0, internal standard), and He (PanGas, purity 5.0, carrier gas) were fed to ensure that the HCl concentration would not exceed 1 vol%. The catalyst was loaded between two plugs of quartz wool in a quartz capillary reactor cell (inner diameter = 0.8 mm), and placed inside an EPR quartz tube, serving as a reactor cell (inner diameter = 2.8 mm, Wilmad). The EPR tube was housed at the center of a homemade water-cooled high-temperature resonator,[8] which was installed into a CW EPR spectrometer (Bruker EMX) operating at X-band frequencies. Prior to feeding the reactant mixtures (0.86 vol% C_2H_2, 0.95 vol% HCl, 1 vol% Ar, 97.19 vol% He) at a total volumetric flow rate of F_T = 95 cm^3 min^{-1}, the catalysts (m_{cat} = 10 mg) were heated under He flow to the desired temperature (T = 473 K). CW-EPR scans were collected during the whole process. Pulse EPR experiments were conducted at commercial Bruker Elexsys E580 spectrometer in a dielectric ER 4118X-MD5 resonator and a home-built Q-band spectrometer (microwave frequency = 34.5-34.8 GHz) in a resonator for oversized tubes (outer diameter = 3 mm).[9] The measurement temperature was set to 40 K. If not stated otherwise, the pulse lengths were set to $t_{\pi/2}$ = 16 ns and t_{π} = 32 ns. Echo-detected field-sweep (EDFS) EPR spectra were recorded as the intensity of primary echo (π/2 - *t* - π - *t* - echo) versus the magnetic field. Delay time (*t*) was set to 0.3 μs. The Hahn echo decay was measured as the intensity of primary echo versus delay *t*. A three-pulse experiment analogous to five-pulse intermolecular hyperfine relaxation-induced dipolar modulation enhancement (ih-RIDME) was performed with variable delay *t* and mixing time (T_{mix}) as π/2 - *t* - π/2 - T_{mix} - π/2 - *t* - echo.[10,11] The delay *t* was incremented in the range 0.2 μs to 4.2 μs. The length of the mixing block was set to 15, 30, 60, 120, 240 μs. For each sample, the ih-RIDME-analog

traces were divided by the reference trace (T_{mix} = 15 μs) and globally fitted assuming a model-free distribution of local proton densities.[12] Furthermore, 2D hyperfine sublevel correlation (HYSCORE) EPR spectroscopy was performed at 50 K, at the field corresponding to signal maximum, on a Bruker EleXsys E580 spectrometer using a split ring resonator (ER4118X-MS3) equipped with an Oxford helium (CF935P) cryostat and operating at a frequency of approximately 9.4 GHz.

Volumetric chemisorption of acetylene was performed in a Micromeritics 3Flex Chemi instrument at 303 K to quantify the amount of acetylene chemisorbed ($V_{C_2H_2}$).

Temperature-programmed desorption (TPD) analysis was performed in a Micromeritics Autochem II 2920 analyzer equipped with a thermal conductivity detector (TCD) and a Pfeiffer Vacuum OmniStar GSD 320 O mass spectrometer. The samples (0.1 g) were loaded into a U-shaped quartz micro-reactor and pre-dried (T = 373 K, heating rate = 10 K min^{-1}, hold time = 20 min, flow rate = 20 cm^3 min^{-1}) in He. Thermal decomposition analysis of the carbon nitride support was initiated by increasing the temperature (T = 313 K, heating rate = 5 K min^{-1}, flow rate = 20 cm^3 min^{-1}) in He while monitoring the formed chemical compounds by mass spectrometry. TPD analysis of C_2H_2 over the carbon nitride support were conducted by cooling the catalyst to 303 K, which was subsequently saturated with a flow of the probe molecule (T = 303 K, hold time = 20 min, F_T = 20 cm^3 min^{-1}). After purging (T = 303 K, hold time = 20 min, F_T = 20 cm^3 min^{-1}, flowing He), the desorption was initiated by increasing the temperature (T = 303 K, heating rate = 5 K min^{-1}, flowing He) while monitoring the desorbed products by mass spectrometry.

Thermogravimetric analysis (TGA) was performed using a Linseis STA PT1600 system. TGA of the as-prepared catalysts and after use in acetylene hydrochlorination was carried out in diluted oxygen (20 vol% O_2/Ar, 100 cm^3 min^{-1}), heating the samples (amount fixed to 20 mg) from 298 to 1073 K at 10 K min^{-1}.

1.3. Catalyst evaluation

The hydrochlorination of acetylene to vinyl chloride (VCM) was evaluated at atmospheric pressure in a continuous-flow fixed-bed micro-reactor, described elsewhere.[3] The gases C_2H_2 (PanGas, purity 2.6), HCl (Air Liquide, purity 2.8, anhydrous), Ar (PanGas, purity 5.0, internal standard), and He (PanGas, purity 5.0, carrier gas), were fed using digital mass-flow controllers (Bronkhorst) to the mixing unit, equipped with a pressure indicator. A quartz micro-reactor of 8 mm internal diameter was loaded with the catalyst (m_{cat} = 0.25 g) and placed in a home-made electrical oven. A K-type thermocouple fixed in a co-axial quartz thermowell with the tip positioned in the center of the catalyst bed was used to control the temperature during the reaction. Prior to testing, the catalyst was heated in a He flow to the desired bed temperature (T = 433-473 K) and allowed to stabilize for at least 15 min before the reaction mixture (40 vol% C_2H_2, 44 vol% HCl, and 16 vol% Ar) was fed at a total volumetric flow rate of F_T = 7.5 cm^3 min^{-1}. Reaction kinetics of acetylene hydrochlorination was studied over Pt_{SA}/ECN at conversion levels <20% with a catalyst mass of m_{cat} = 0.1 g, a total flow of F_T = 15 cm^3 min^{-1}, and reactant concentrations of 10-30 vol%, balanced in He, to determine the partial reaction order of the

reactants ($n_{C_2H_2}$, n_{HCl}). Carbon-containing compounds (C_2H_2 and C_2H_3Cl) and Ar were quantified on-line *via* a gas chromatograph equipped with a GS-Carbon PLOT column coupled to a mass spectrometer (GC-MS, Agilent, GC 7890B, Agilent MSD 5977A). Since VCM was the only product detected in all our tests, the catalytic activity is presented as the yield of VCM, Y_{VCM}, calculated according to Eq. 1,

$$Y_{VCM},\% = \frac{n_{VCM}^{outlet}}{n_{C_2H_2}^{inlet}} \times 100 \qquad \text{Eq. 1}$$

where n_{VCM}^{outlet} and $n_{C_2H_2}^{inlet}$ denote the molar flows of VCM and C_2H_2 at the reactor outlet and inlet, respectively. The overall reaction rate (r) was determined according to Eq. 2,

$$r,\ \mathrm{mol_{VCM}\ s^{-1}\ g_{cat}^{-1}} = \frac{n_{VCM}^{outlet}}{m_{cat}} \qquad \text{Eq. 2}$$

where m_{cat} denotes the catalyst mass. The error of the carbon balance, ε_C, determined using Eq. 3, was less than 10% in all experiments, *i.e.*, the carbon mass balance was closed at ≥90%.

$$\varepsilon_C,\% = \frac{n_{C_2H_2}^{inlet} - \left(n_{C_2H_2}^{outlet} + n_{VCM}^{outlet}\right)}{n_{C_2H_2}^{inlet}} \times 100 \qquad \text{Eq. 3}$$

After the tests, the reactor was quenched to room temperature (ca. 298 K) in He flow and the catalyst was retrieved for further characterization. The deactivation constants, k_D, were derived *via* an exponential regression of the catalytic data collected over 24 h on stream. To gain deeper insights into the reaction kinetics and catalyst dynamic behavior, time-resolved product analysis was conducted with a Pfeiffer Vacuum OmniStar GSD 320 O mass spectrometer.

1.4. Evaluation of mass and heat transfer limitations

The evaluation of the dimensionless moduli based on the criteria of Carberry,[13] Mears,[14] and Weisz-Prater,[15] confirmed that all the catalytic tests were performed in the absence of mass and heat transfer limitations. The Carberry criterion (*Ca*) was used to evaluate external mass transfer limitations according to Eq. 4,

$$Ca = \frac{r_{v,obs}}{a' k_f c_b} < \frac{0.05}{|n|} \qquad \text{Eq. 4}$$

where k_f is the mass transfer coefficient (estimated at a minimum value of 0.01 m s^{-1}), c_b is the bulk concentration of acetylene (17.6 mol m^{-3}), n is the reaction order, while $r_{v,obs}$ and a' denote the reaction rate and the specific particle area, which are derived *via* Eq. 5 and Eq. 6, respectively,

$$r_{v,obs} = \frac{n_{VCM}^{outlet}}{V_{cat}} \qquad \text{Eq. 5}$$

$$a' = \frac{1}{L} = \frac{A_p}{V_p} = \frac{4}{d_p} \qquad \text{Eq. 6}$$

where L, A_p, V_p, and d_p are the characteristic length, the area, the volume, and the diameter of the particle. To assess extra-particle temperature gradients (ΔT_e),[13] Eq. 7 was applied,

$$\Delta T_e = \beta_e Ca = \frac{-\Delta H_r k_f c_b Ca}{h T_b} \qquad \text{Eq. 7}$$

where β_e denotes the external Prater number, T_b the temperature in the bulk phase (473 K), h the heat transfer coefficient (estimated at a minimum value of 10 J m^{-2} s^{-1} K^{-1}), and $-\Delta H_r$ the reaction enthalpy (99.3 kJ mol^{-1}). Internal mass transfer limitations were evaluated using the Weisz-Prater criterion (Φ),[14] according to Eq. 8,

$$\Phi = \frac{r_{v,obs} L^2}{D_{eff} c_s}\left(\frac{n+1}{2}\right) < 1 \qquad \text{Eq. 8}$$

where L is the characteristic length (0.2 mm), c_s is the surface concentration ($c_s \approx c_b$ in the absence of external mass transfer limitations), and the effective diffusion coefficient, which can be derived *via* Eq. 9,

$$D_{eff} = \frac{\varepsilon}{\tau} D_{C_2H_2,\,HCl} \qquad \text{Eq. 9}$$

where τ is the tortuosity factor (estimated at 3), ε is the particle porosity (estimated at 0.2), and $D_{C_2H_2,HCl}$ is the molecular diffusion coefficient (1.96·10^{-5} m^2 s^{-1}). Intra-particle temperature gradients (ΔT_i) can be calculated using Eq. 10,[13]

$$\Delta T_i = \beta_i T_s = \frac{-\Delta H_r D_{eff} c_b}{\lambda_{eff}} T_s \qquad \text{Eq. 10}$$

where β_i denotes the internal Prater number and λ_{eff} the effective thermal conductivity (estimated at 0.5 W K^{-1} m^{-1}).

The results for Pt_{SA}/ECN are given in **Supplementary Table 11**. The Carberry criterion (Eq. 4) is met in all catalytic tests while the external temperature difference is negligible (<0.01 K), indicating the absence of external mass and heat transfer limitations, respectively. Finally, the temperature gradient within the catalyst particle is negligible for each case, indicating the absence of internal heat transfer limitations.

1.5. Computational methods

DFT simulations were performed using the Vienna Ab initio Simulation Package (VASP, **Figure 7**, **Supplementary Figures 5** and **20**, **Supplementary Tables 9** and **19**).[16,17] The generalized gradient approximation of the Perdew-Burke-Ernzerhof functional was used to obtain the exchange-correlation energies with dispersion contributions introduced *via* Grimme's DFT-D3 approach.[18,19] Core electrons were treated using projector augmented wave PAW for the core and plane waves with a cut-off energy of 450 eV for the valence were chosen with spin polarization allowed when needed.[20,21] For all investigated systems, structures were relaxed using convergence criteria of 10^{-4} and 10^{-5} eV for the ionic and electronic steps, respectively. Dipole correction along the z-axis were employed for slab models.[22] ECN was modeled as a heptazine 2×2 supercell of four layers with the bottom one fixed to the bulk

configuration, considering 6 coordination sites: (*i*) pyridinic N-atoms in the triazine cavity (N_{2C}), (*ii*) graphitic N-atoms in the heptazine unit (N_{3C}), (*iii*) graphitic N-atoms linking three heptazine units ($N_{3C,link}$), and (*iv*-*vi*) their corresponding vacancies. The ECN-supported Pt SAC was modeled by placing $PtCl_2$ moieties at the center of the distinct ECN coordination sites. Transition states were located by using Climbing Image Nudge Elastic Band (CI-NEB). They were verified through a frequency numerical analysis with a step size of ± 0.015 Å, and confirmed by the presence of only one imaginary frequency.[23] During the proposed reaction mechanism for the interaction with C_2H_2, additional entropic contributions arise due to the increased flexibility of the scaffold and the potential partial migration of Cl ligands. Notably, configurational entropy poses challenges for analysis and quantification, limiting the evaluation of these contributions. Consequently, the reaction profile is primarily presented in terms of potential energy, with entropic corrections applied only to the release of gas-phase molecules, specifically NH_3 in this case. In instances where these corrections are utilized, we can infer that similar contributions from the other entropic terms are implicitly accounted for. The structural parameters (*g* and *A* tensors, **Supplementary Figure 9**, **Supplementary Table 6**) for the analysis of experimental CW-EPR and 2-pulse ESEEM spectra were determined with Kohn-Sham DFT, using a B3LYP functional with a spin-unrestricted shell, a DGTZVP basis set for triazine radicals and a 6-31G for extended carbon nitride systems in the Gaussian and Orca software.[24,25]

2. Supplementary tables

Supplementary Table 1. Surface area, as determined by nitrogen sorption, and initial activity in acetylene hydrochlorination of selected carbon nitride supports.

Support	S_{BET} / m^2 g^{-1}	$Y_{VCM,0}$[a] / %
CN	11	1
ECN	329	3
ppCN	7	1
EppCN	34	3
LMO	4	0
ELMO	16	1

[a]Initial yield of vinyl chloride monomer after 1 h on stream.

Supplementary Table 2. Surface composition of selected carbon nitride-supported Pt SACs as determined by XPS.

Catalyst	C / at%	N / at%	Cl / at%
Pt_{SA}/CN	45.0	54.5	0.5
Pt_{SA}/ECN	44.3	55.5	0.3
Pt_{SA}/ECN-24h[a]	45.7	53.3	1.0

[a]Catalyst after use in acetylene hydrochlorination for 24 h.

Supplementary Table 3. Fitting parameters derived from the Pt 4*f* XPS spectra of selected carbon nitride-supported Pt SACs.

Catalyst	Pt^{0a}			Pt^{IIa}			Pt^{IVa}		
	Position / eV	FWHM[b] / eV	Area / %	Position / eV	FWHM[b] / eV	Area / %	Position / eV	FWHM[b] / eV	Area / %
Pt_{SA}/CN	71.2	-	-	72.6	1.6	67	73.8	2.5	33
Pt_{SA}/ECN	71.2	-	-	72.6	1.6	62	73.5	2.2	38
Pt_{SA}/ECN-24h[c]	71.2	-	-	72.6	1.8	71	73.5	2.5	29

[a]Assignment based on reference values.[3,26] [b]FWHM: full width at half maximum. [c]Catalyst after use in acetylene hydrochlorination for 24 h.

Supplementary Table 4. Fitting parameters derived from the Cl 2*p* XPS spectra of selected carbon nitride-supported Pt SACs.

Catalyst	Cl–C[a]			Cl–Pt[a]		
	Position / eV	FWHM[b] / eV	Area / %	Position / eV	FWHM[b] / eV	Area / %
Pt_{SA}/CN	200.6	-	-	198.1	1.7	100
Pt_{SA}/ECN	200.6	-	-	197.8	2.0	100
Pt_{SA}/ECN-24h[c]	200.6	1.9	14	197.8	2.2	86

[a]Assignment based on reference values.[26] [b]FWHM: full width at half maximum. [c]Catalyst after use in acetylene hydrochlorination for 24 h.

Supplementary Table 5. Experimental EPR parameters for Pt_{SA}/ECN (**Figure 2**).

Component	Defect[a]	g_{xx}[b]	g_{yy}[b]	g_{zz}[b]
1	N_{3C}-vacancy	2.0044	2.0044	2.0044
2	N_{2C}-vacancy	2.0062	2.0062	2.0012
3	unidentified	2.0050	1.9998	1.9998

[a]Paramagnetic defect associated with each spectral component.
[b]Principal values of the *g* tensor.

Supplementary Table 6. DFT-calculated EPR parameters for tri-heptazine systems, containing a single N_{3C}-vacancy or N_{2C}-vacancy.

Vacancy	Atom[a]	g_{xx}[b]	g_{yy}[b]	g_{zz}[b]	A_{iso}[c]	T_{xx}[d]	T_{yy}[d]	T_{zz}[d]
N_{2C}		2.00374	2.00475	2.00174				
	N_1				8.44711	−6.007	−5.809	11.815
	N_2				9.46091	−6.365	−6.316	12.681
	N_3				3.59436	−3.240	−3.096	6.336
	N_4				12.51299	−8.300	−8.248	16.548
	N_5				11.86798	−8.161	−7.888	16.050
N_{3C}		2.00184	2.00158	2.00275				
	N_1				−5.81217	−4.308	1.217	3.091
	N_2				−5.13291	−4.166	1.217	2.949
	N_3				−4.24704	−3.235	0.842	2.393
	N_4				−4.31574	−3.290	0.862	2.428
	N_5				−3.80781	−2.927	0.717	2.209
	N_6				−3.78734	−2.876	0.684	2.192

[a]N-atoms surrounding the N-vacancy. Principal values of [b]the *g* tensor, [c]the isotropic hyperfine coupling of the *A* tensor, and [d]the anisotropic hyperfine coupling of the *T* tensor. The calculated *g* values are consistently lower than experimental ones (**Supplementary Table 5**), as the actual carbon nitride fragments are much larger than the simulated ones.

Supplementary Table 7. Fitting parameters derived from the Pt L_3 edge EXAFS spectra of selected carbon nitride-supported Pt SACs.

Catalyst	Coordination	CN[a] / -	σ^{2b} / Å^2	R^c / Å	R_f^d / -
Pt_{SA}/ECN	Pt–N/C	2.1 ± 0.2	0.005 ± 0.002	2.01 ± 0.02	0.006
	Pt–Cl	3.2 ± 0.2	0.002 ± 0.002	2.31 ± 0.02	
Pt_{SA}/ECN-1h[e]	Pt–N/C	0.8 ± 0.2	0.005 ± 0.002	2.03 ± 0.02	0.005
	Pt–Cl	3.3 ± 0.2	0.002 ± 0.002	2.32 ± 0.02	
Pt_{SA}/ECN-3h[e]	Pt–N/C	0.7 ± 0.2	0.005 ± 0.002	2.03 ± 0.02	0.004
	Pt–Cl	3.4 ± 0.2	0.002 ± 0.002	2.31 ± 0.02	
Pt_{SA}/ECN-24h[e]	Pt–N/C	1.1 ± 0.2	0.005 ± 0.002	2.00 ± 0.02	0.007
	Pt–Cl	2.6 ± 0.2	0.002 ± 0.002	2.32 ± 0.02	
Pt_{SA}/ECN-HCl[f]	Pt–N/C	0.7 ± 0.2	0.005 ± 0.002	2.01 ± 0.02	0.003
	Pt–Cl	5.2 ± 0.2	0.002 ± 0.002	2.32 ± 0.02	
Pt_{SA}/ECN-C_2H_2[g]	Pt–N/C	1.8 ± 0.2	0.005 ± 0.002	1.86 ± 0.02	0.012
	Pt–N/C	3.3 ± 0.2	0.005 ± 0.002	2.04 ± 0.02	
	Pt–Cl	0.6 ± 0.2	0.002 ± 0.002	2.34 ± 0.02	

[a]Coordination number. [b]Debye-Waller factor. [c]Coordination shell distance. [d]*R*-factor. [e]Catalyst after use in acetylene hydrochlorination, denoted as "-*Y*h". *Y* = time on stream in h. [f]Catalyst after exposure to only HCl for 1 h. [g]Catalyst after exposure to only C_2H_2 for 1 h.

Supplementary Table 8. Parameters of bimodal distributions of local proton densities in Pt_{SA}/ECN, as prepared and after use in acetylene hydrochlorination, as determined from fitting the ih-RIDME-analog experimental traces (**Supplementary Figure 17**).

Catalyst	$Mean_1$[a] / M	Std_1[b] / M	$Area_1$[c] / -	$Mean_2$[a] / M	Std_2[b] / M	$Area_2$[c] / -
Pt_{SA}/ECN	17.0	0.6	0.11	31.3	1.6	0.89
Pt_{SA}/ECN-1h[d]	23.4	0.7	0.13	44.5	7.1	0.87
Pt_{SA}/ECN-3h[d]	22.1	0.6	0.13	42.7	1.9	0.87

[a]Mean value, [b]standard deviation, and [c]area of the first and second components, at low and high local proton concentration densities respectively, as indicated by the pedices. [d]Catalyst after use in acetylene hydrochlorination, denoted as "-*Y*h". *Y* = time on stream in h.

Supplementary Table 9. Bader charges (in $|e^{-}|$) of pristine $PtCl_2$ and upon HCl adsorption, stabilized on one or two N_{2C} sites in the ECN support as indicated by XAS analysis (**Supplementary Table 7**).

Metal species	Bader charge
$PtCl_2$–$2N_{2C}$	0.67
$PtCl_3$–N_{2C} + $H^{\#a}$	0.55
$PtCl_3$–$2N_{2C}$ + $H^{\#a}$	0.85

[a]Upon HCl adsorption on the Pt site, H is transferred to a site, #, in the carbon nitride support.

Supplementary Table 10. Adsorption Gibbs free energy (G_{ads} in eV) of C_2H_2 on distinct N-atoms and N-vacancies in the ECN support for $PtCl_2$ (**Supplementary Figure 31**), in pristine state and after HCl-induced depolymerization by $N_{3C,link}$ protonation.

Site[a]	Pristine	After depolymerization
N_{2C}-atom	−0.03	0.96
N_{3C}-atom	−0.07	2.24
$N_{3C,link}$-atom	0.09	0.43
N_{2C}-vacancy	−3.63	-
N_{3C}-vacancy	−1.04	-
$N_{3C,link}$-vacancy	−5.81	-

[a] $G_{ads} = G(C_2H_2^*) - G(ECN) - G(C_2H_2,gp)$. $G(C_2H_2^*)$ is the Gibbs free energy of the binding site with adsorbed C_2H_2, G(ECN) is the Gibbs free energy of the binding site in the ECN support, and $G(C_2H_2, gp)$ is the Gibbs free energy of C_2H_2 gas-phase.

Supplementary Table 11. Results for mass and heat transfer limitation criteria.

Catalyst	$r_{v,obs}$ / mol_{VCM} s^{-1} m_{cat}^{-3}	Ca[a] / -	ΔT_e[b] / K	Φ[c] / -	ΔT_i[d] / K
Pt_{SA}/ECN	2.9	0.0014	0.005	0.07	0.74

[a]Carberry criterion. [b]Extra-particle temperature gradient. [c]Weisz-Prater criterion. [d]Intra-particle temperature gradient.

3. Supplementary figures

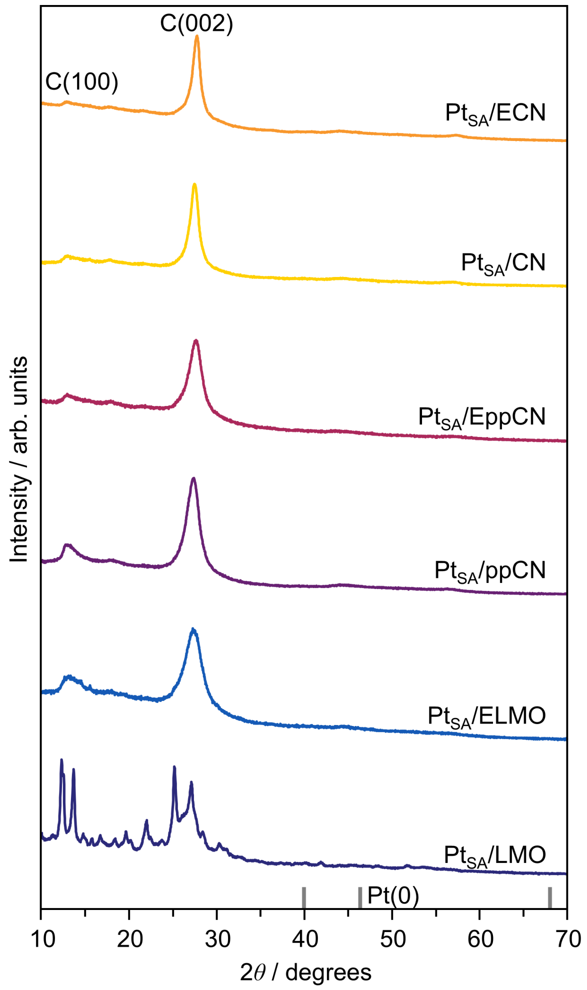

Supplementary Figure 1. XRD patterns of carbon nitride-supported Pt SACs. Diffraction peaks of metallic platinum are indicated by vertical gray bars.

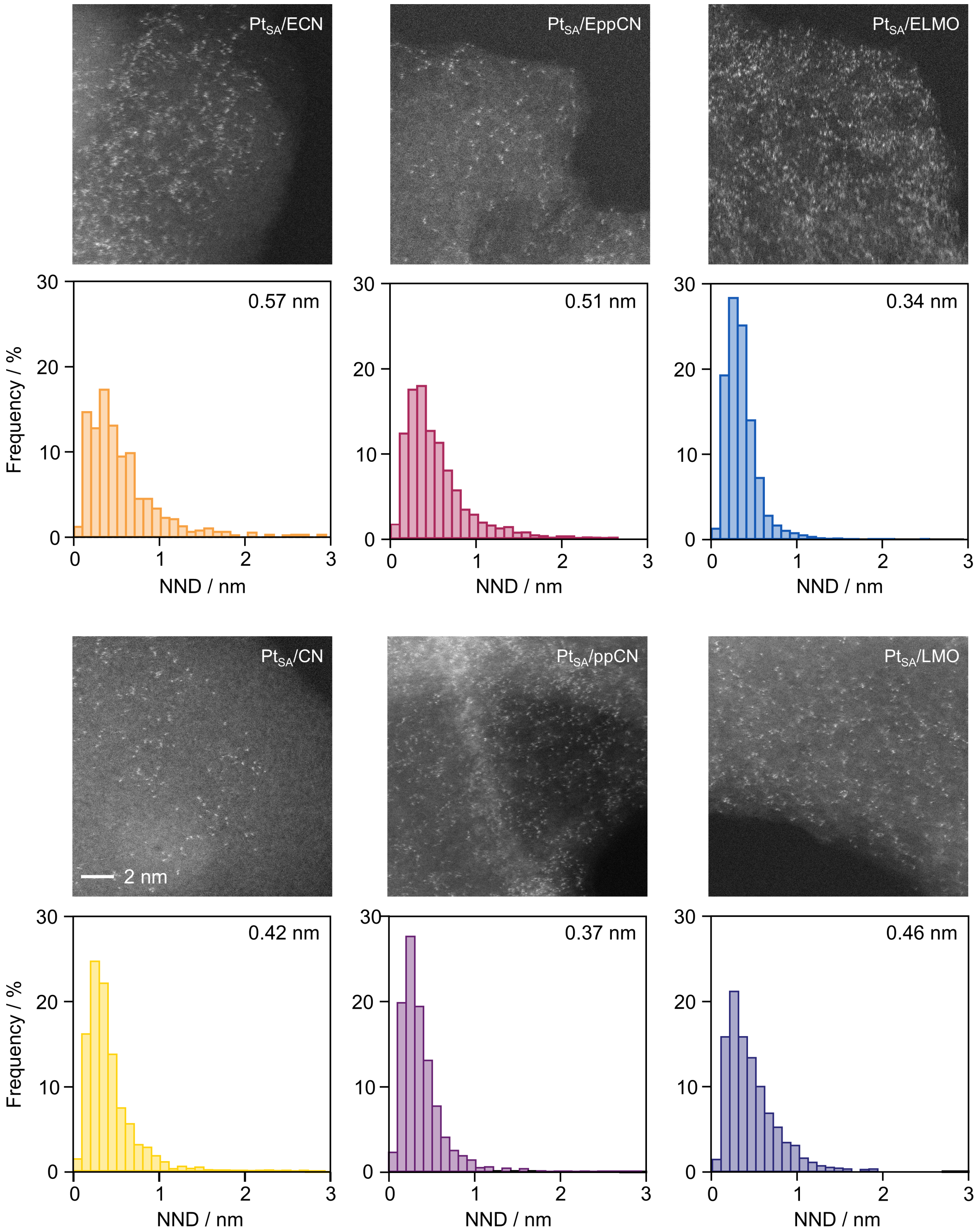

Supplementary Figure 2. HAADF-STEM images (top) of as-prepared carbon nitride-supported Pt SACs, together with plots of nearest-neighbor distributions, NND, (bottom) between metal centers and their mean value performed using an atom detection inference pipeline, accessible through the web app https://huggingface.co/spaces/nccr-catalysis/atom-detection. Scale bar applies to all images.

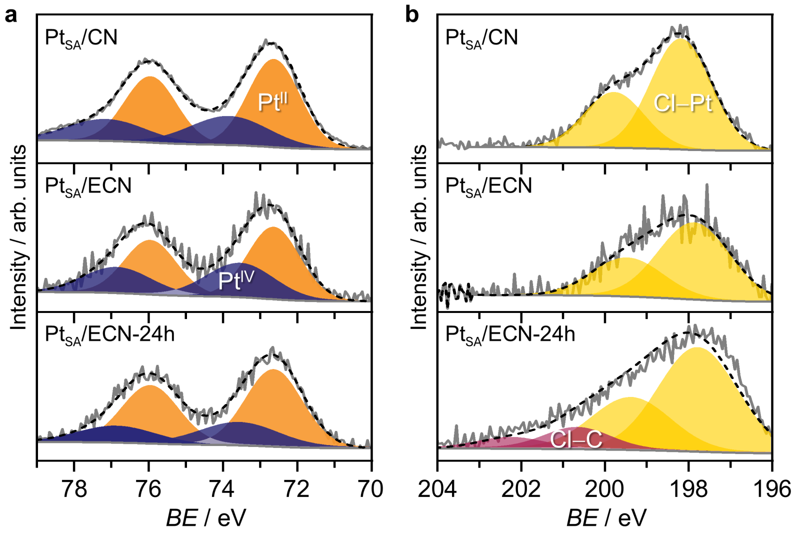

Supplementary Figure 3. a, Pt 4*f* and **b,** Cl 2*p* experimental and fitted XPS spectra of selected carbon nitride-supported Pt SACs, as-prepared and after use in acetylene hydrochlorination.

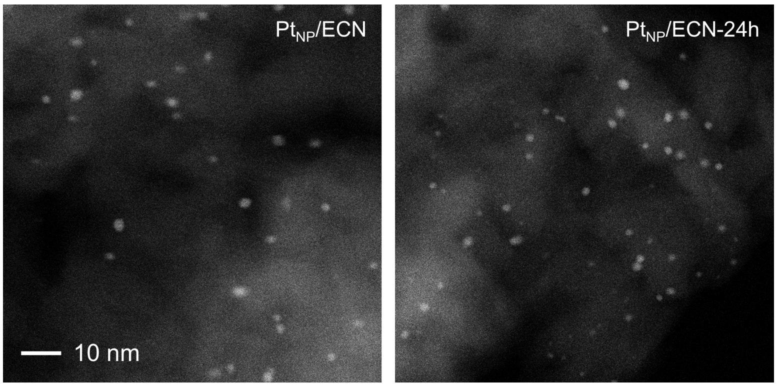

Supplementary Figure 4. HAADF-STEM images of Pt_{NP}/ECN, as-prepared and after use in acetylene hydrochlorination. Scale bar applies to all images.

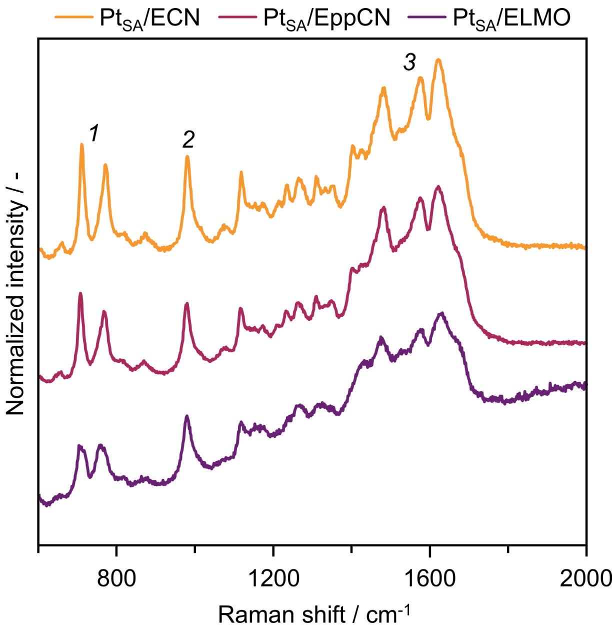

Supplementary Figure 5. Raman spectra of selected carbon nitride-supported Pt SACs. Three contributions are identified.[27] *1*: in-plane bending of CNC linkages. *2*: symmetric breathing of N-atoms within heptazine units. *3*: symmetric stretching of C–N bonds.

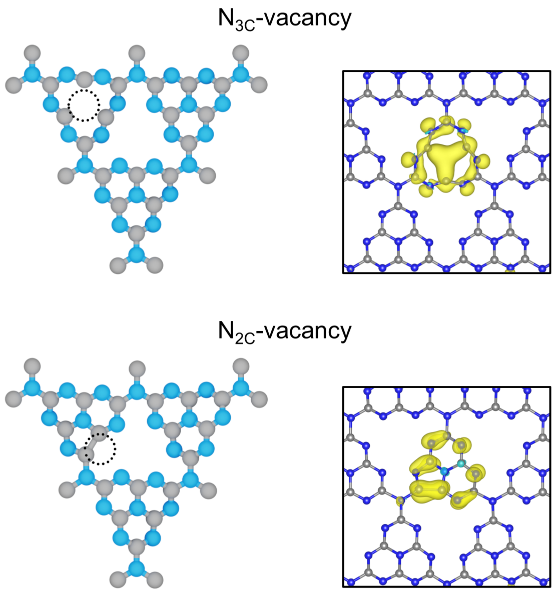

Supplementary Figure 6. Structural representation of the N_{2C}- and N_{3C}-vacancies (left), together with the associated delocalization of the spin density across the heptazine unit (right, plotted using the VESTA software).[28] Isosurface value was set to $\rho = 002$ |e| $Å^{-3}$. Color code: blue N and gray C (atoms); yellow positive values and light blue negative values (spin density).

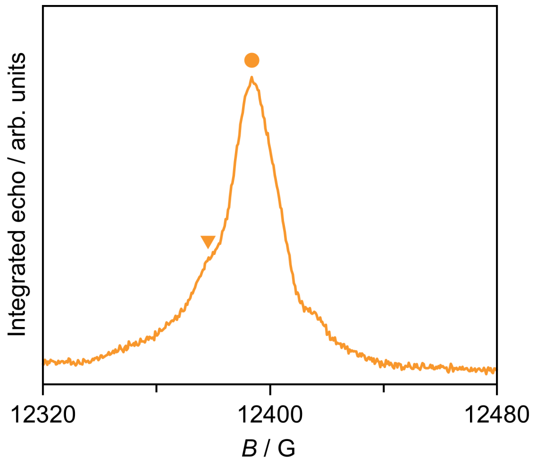

Supplementary Figure 7. Q-band EDFS EPR spectrum of Pt_{SA}/ECN, showing the field positions at which the 2-pulse ESEEM spectra reported in **Supplementary Figure 8** (*vide infra*) are recorded, corresponding to the maxima of N_{3C}-vacancy (circle) and N_{2C}-vacancy (inverted triangle).

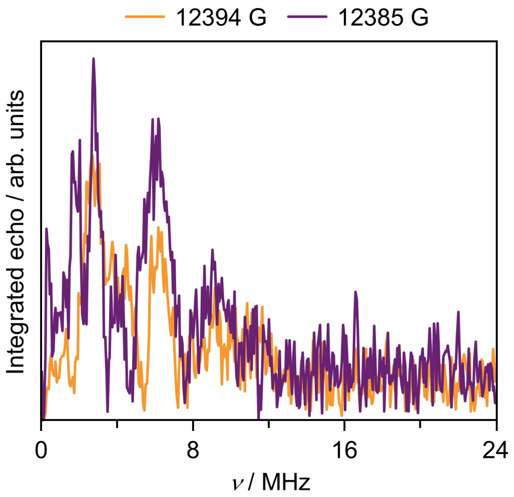

Supplementary Figure 8. Q-band 2-pulse ESEEM EPR spectra of Pt_{SA}/ECN recorded at field positions, corresponding to the maxima of N_{3C}-vacancy (12394 G) and N_{2C}-vacancy (12385 G).

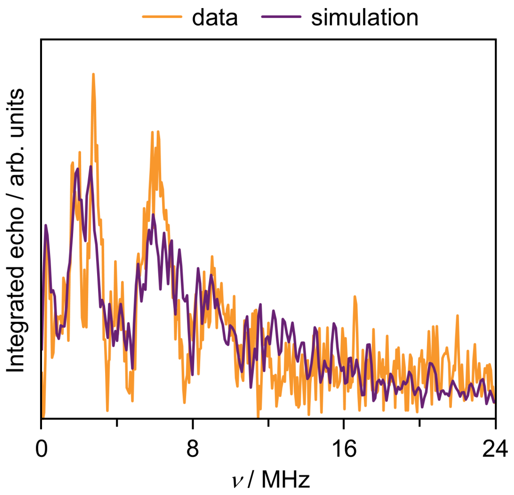

Supplementary Figure 9. Experimental Q-band 2-pulse ESEEM EPR spectrum of Pt_{SA}/ECN with simulation based on DFT-calculated hyperfine couplings, reported in **Supplementary Table 6**.

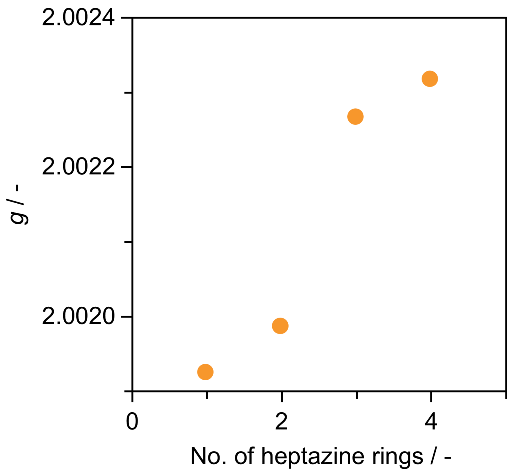

Supplementary Figure 10. DFT-calculated average *g* values for systems with a different number of connected heptazine units, containing one N_{3C}-vacancy.

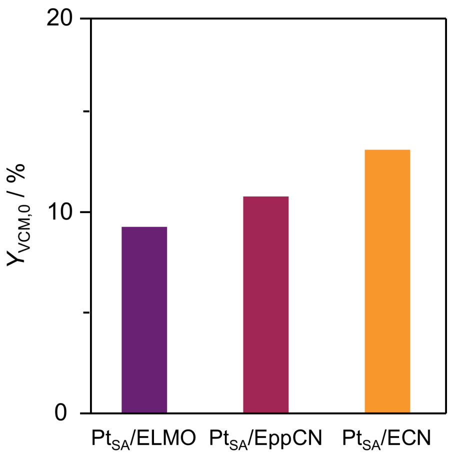

Supplementary Figure 11. Initial activity, expressed as VCM yield after 1 h on stream, $Y_{VCM,0}$, of carbon nitride-supported Pt SACs with constant metal-content-to-surface-area ratio (*i.e.*, metal density, 75 μmol_{Pt} m^{-2}). The catalyst mass, and thus space velocity, was varied to maintain a constant reactant flow rate per metal site. Reaction conditions: $T_{bed} = 473$ K, $F_{tot} = 7.5$ cm^3 min^{-1}, C_2H_2:HCl:Ar = 40:44:16, $W_{cat} = 0.05$-1.06 g.

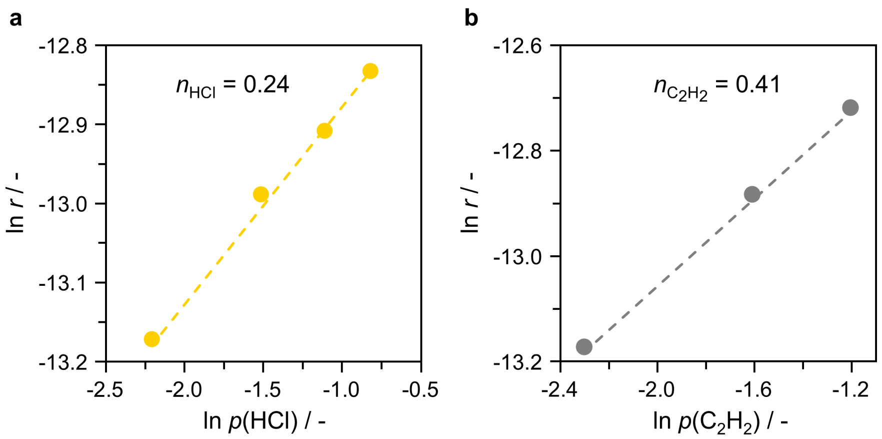

Supplementary Figure 12. Reaction rate over Pt_{SA}/ECN as a function of partial pressure of **a,** HCl and **b,** C_2H_2. Partial reaction orders of HCl, n_{HCl}, and C_2H_2, $n_{C_2H_2}$, correspond to the slope of the fitting lines in **a** and **b**, respectively. Reaction conditions: T = 473 K, m_{cat} = 0.1 g, F_T = 15 cm^3 min^{-1}, $p_{C_2H_2}$ = 0.1 bar in **a**, p_{HCl} = 0.11 bar in **b**.

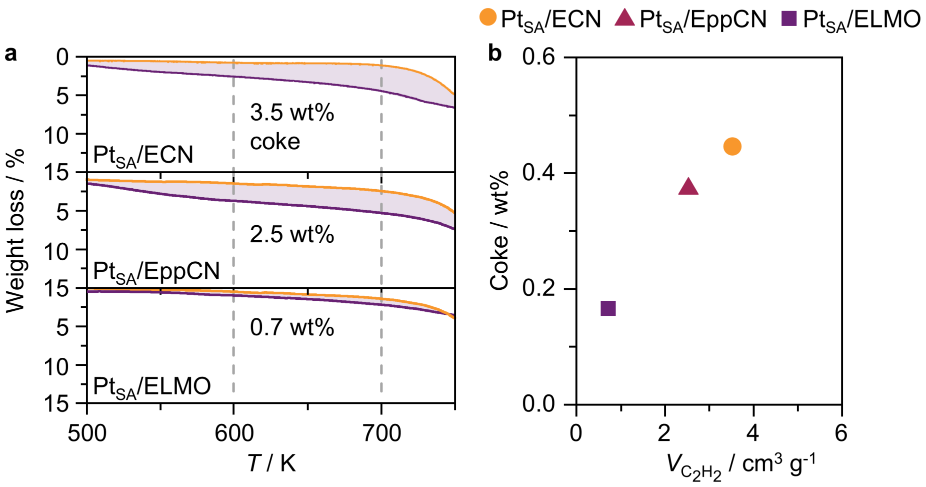

Supplementary Figure 13. a, TGA in 20 vol% O_2/Ar of selected carbon nitride-supported Pt SACs, as-prepared and after use in acetylene hydrochlorination, and **b,** coke amount in the used catalysts as a function of C_2H_2 chemisorption capacity, V_{C2H2} of the bare pristine supports. The difference in weight loss is highlighted by the purple-colored area. Quantification of coke in the used catalysts was performed by integrating the TGA profile in reference to the as-prepared catalysts in the temperature range of 600-700 K.

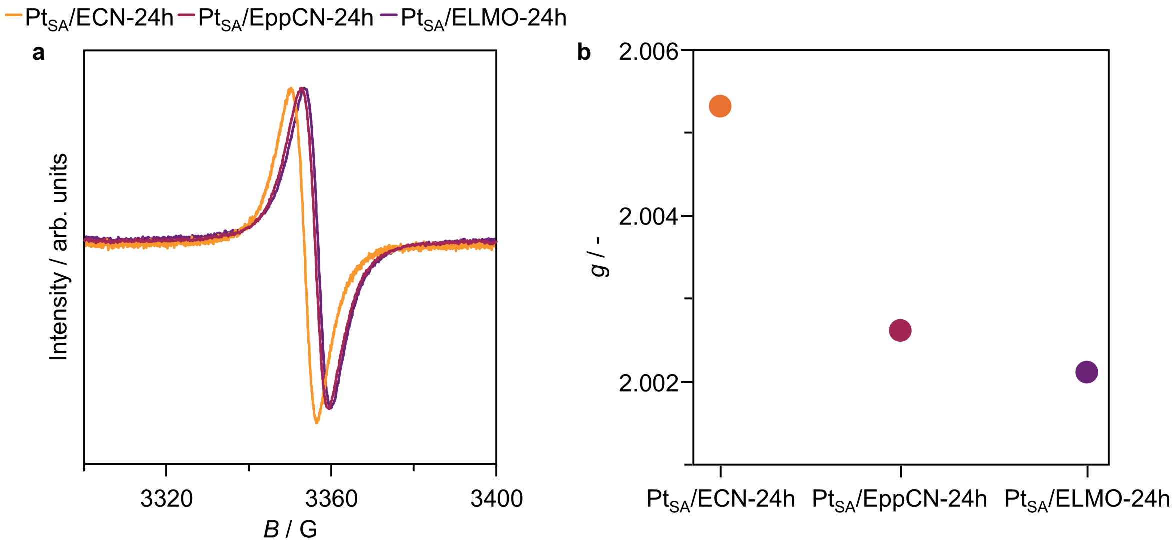

Supplementary Figure 14. a, CW-EPR spectra and **b,** corresponding average *g* factors of selected carbon nitride-supported Pt SACs after use in acetylene hydrochlorination for 24 h.

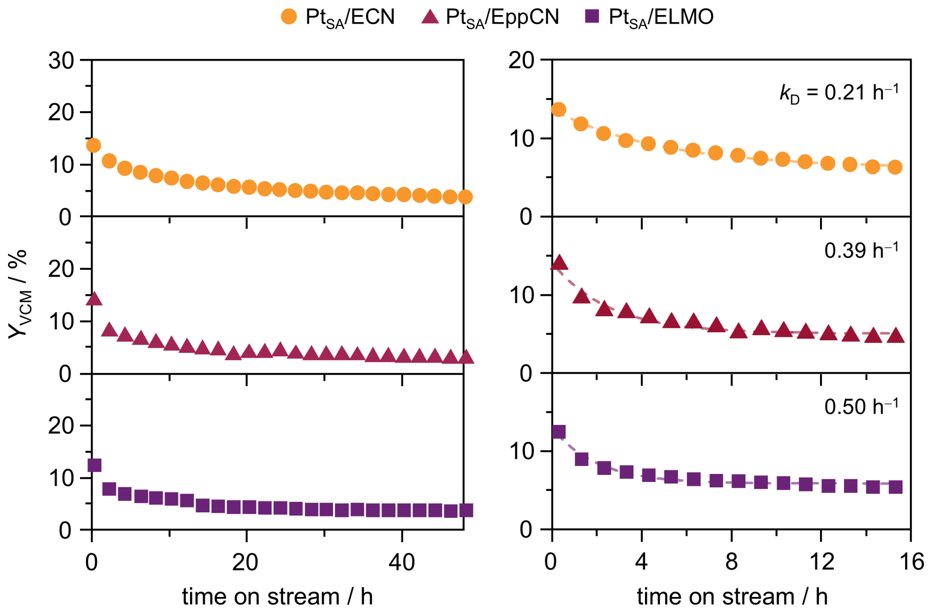

Supplementary Figure 15. Catalytic performance, Y_{VCM}, of selected carbon nitride-supported Pt SACs over time on stream (left) with respective deactivation constants, k_D, as determined by exponential regression (right). The catalyst mass, and thus space velocity, was varied to obtain a comparable VCM yield across the different catalysts. Reaction conditions: $GHSV(C_2H_2) = 81\text{-}650\ h^{-1}$, ($C_2H_2$:HCl:Ar = 40:44:16, $T = 473$ K, $P = 1$ bar.

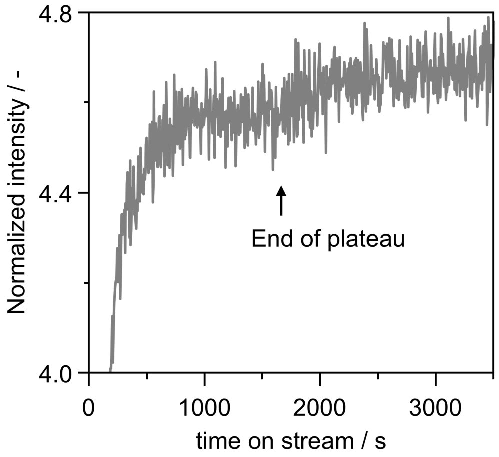

Supplementary Figure 16. Time-resolved product analysis over Pt_{SA}/ECN by mass spectroscopy, showing m/z 26 (C_2H_2) presented in **Figure 4b** on a linear scale.

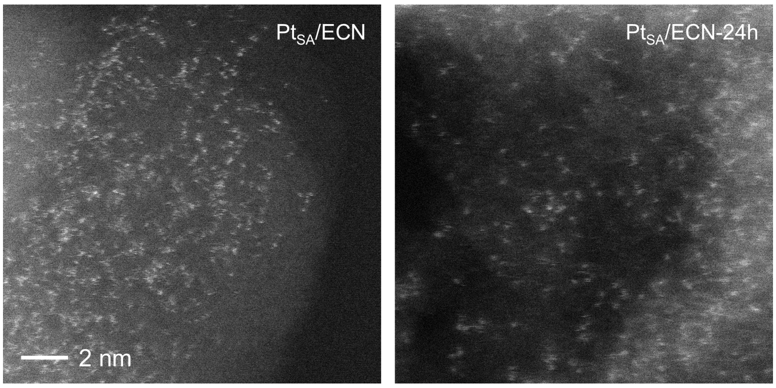

Supplementary Figure 17. HAADF-STEM images of Pt_{SA}/ECN, as-prepared and after use in acetylene hydrochlorination. Scale bar applies to all images.

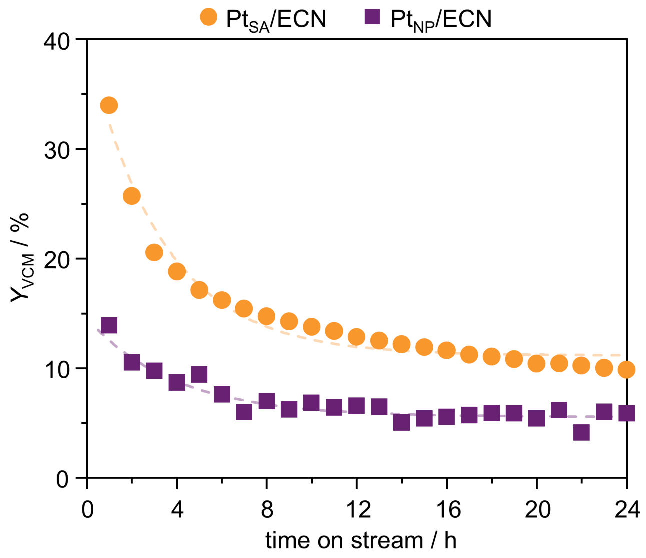

Supplementary Figure 18. Catalytic performance, Y_{VCM}, of Pt single atoms and nanoparticles supported on ECN over time on stream. Reaction conditions: $T_{bed} = 473$ K, $F_{tot} = 7.5$ cm^3 min^{-1}, C_2H_2:HCl:Ar = 40:44:16, $W_{cat} = 0.25$ g.

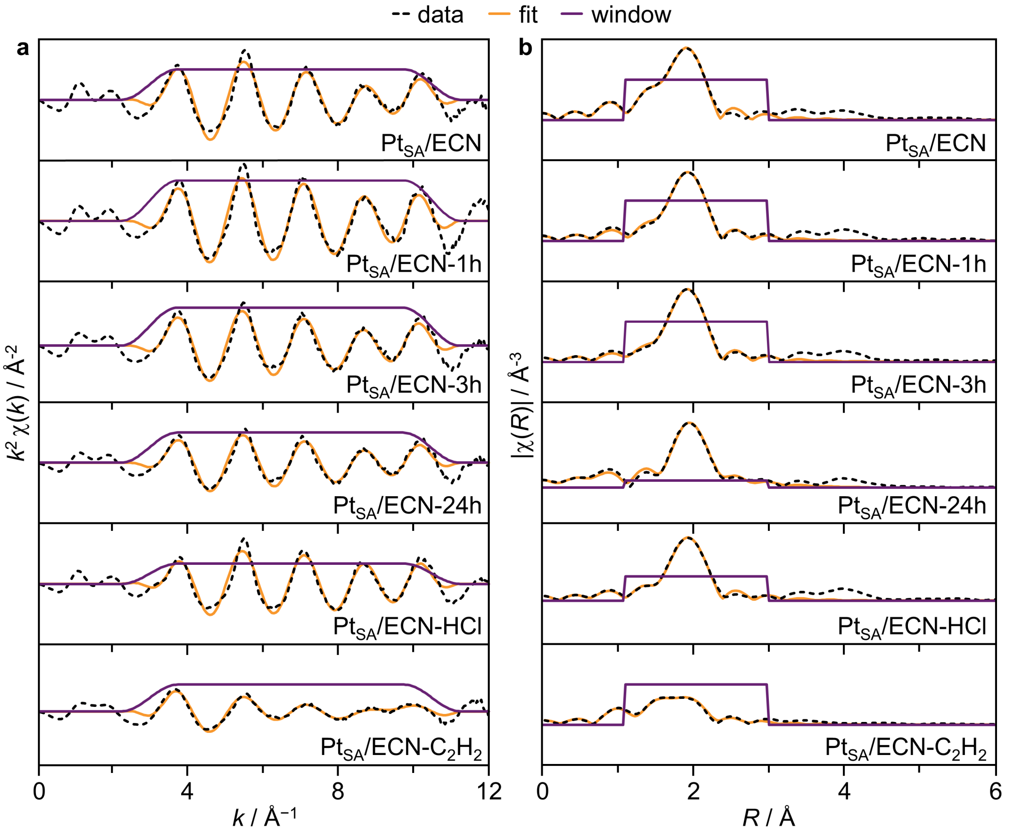

Supplementary Figure 19. a, *k* space and **b,** *R* space of experimental and fitted Pt L_3 edge EXAFS spectra of Pt$_{SA}$/ECN, as prepared, after use in acetylene hydrochlorination and after exposure to HCl only and C_2H_2 only.

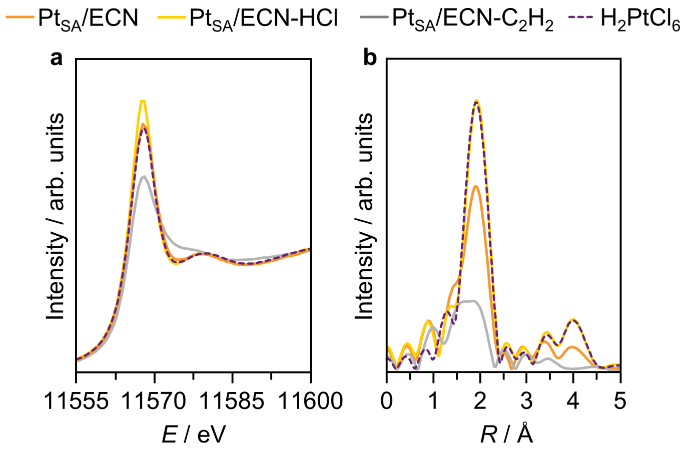

Supplementary Figure 20. Pt L_3 edge **a,** XANES and **b,** EXAFS of Pt_{SA}/ECN, as-prepared and after exposure to HCl only and C_2H_2 only, together with the H_2PtCl_6 reference.

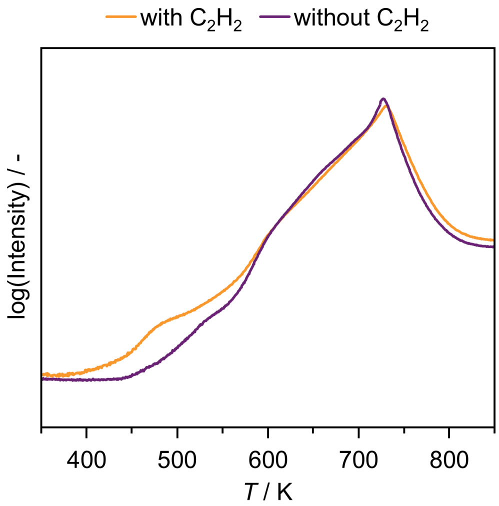

Supplementary Figure 21. Temperature-resolved C_2H_2-TPD-MS analysis over the bare ECN support, after saturation with C_2H_2 and after flowing He in the absence of C_2H_2.

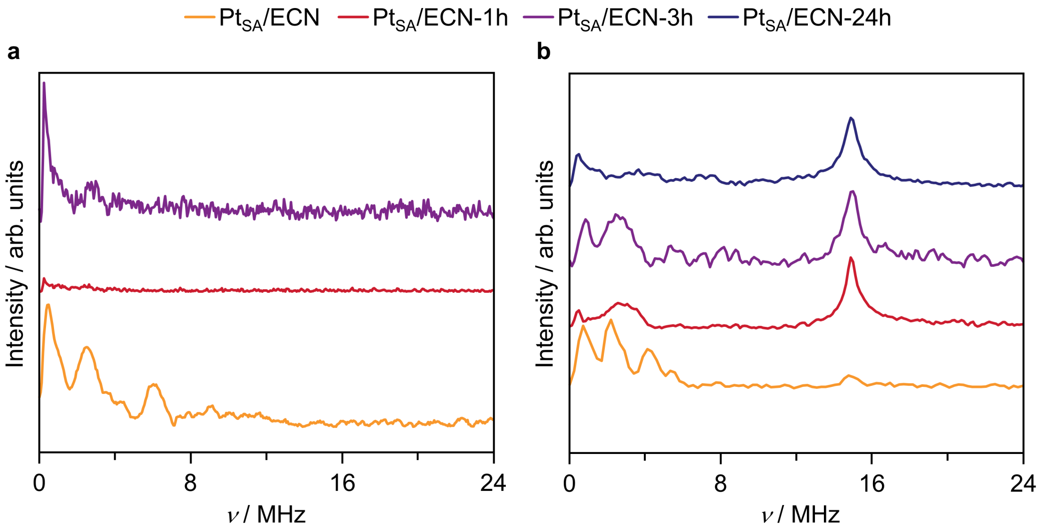

Supplementary Figure 22. a, Q-band and **b,** X-band 2-pulse ESEEM Fourier-transformed EPR spectra of Pt_{SA}/ECN, as prepared and after use in acetylene hydrochlorination.

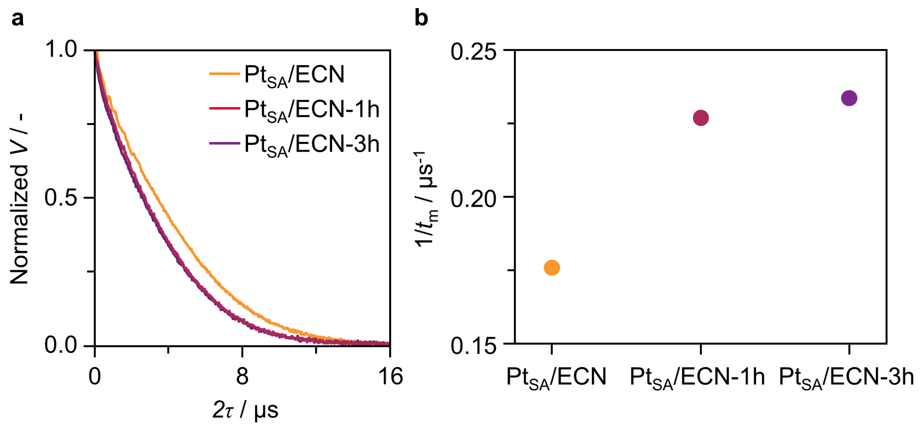

Supplementary Figure 23. a, Normalized experimental EPR-determined Hahn echo decay, *V*, and **b,** inverse phase memory times, t_m, of Pt_{SA}/ECN, as-prepared and after use in acetylene hydrochlorination. The differences in the decay rates reflect different proton density in the vicinity of the paramagnetic defects.

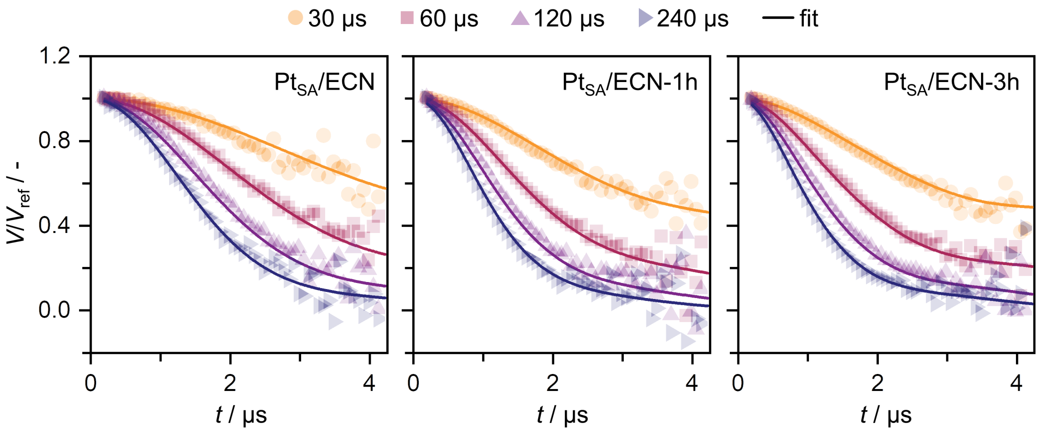

Supplementary Figure 24. Experimental and fitted ih-RIDME-analog EPR traces, V, of different mixing block lengths for Pt_{SA}/ECN, as-prepared and after use in acetylene hydrochlorination, divided by the reference trace at mixing block length of 15 µs, V_{ref}.

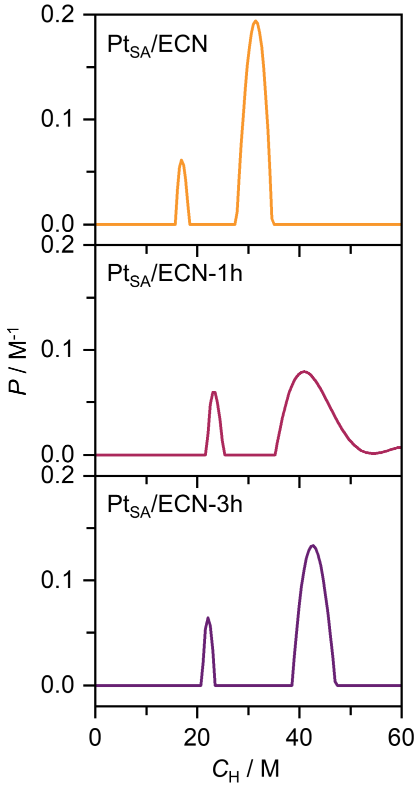

Supplementary Figure 25. Distribution, *P*, of local proton densities, C_H, obtained by fitting the ih-RIDME-analog EPR experimental traces for Pt_{SA}/ECN, as-prepared and after use in acetylene hydrochlorination.

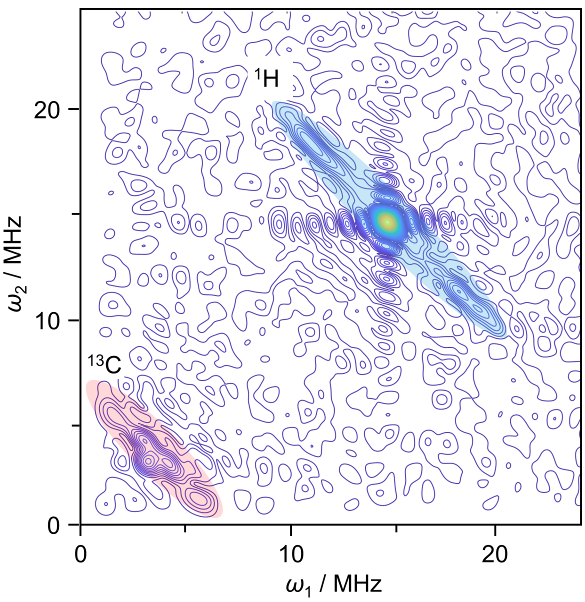

Supplementary Figure 26. X-band 4-pulse HYSCORE EPR spectrum of Pt_{SA}/ECN after use in acetylene hydrochlorination for 24 h.

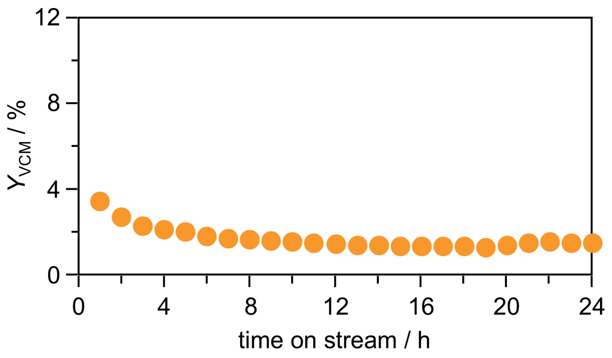

Supplementary Figure 27. Catalytic performance, Y_{VCM}, of bare ECN over time on stream. Reaction conditions: T_{bed} = 473 K, F_{tot} = 7.5 cm^3 min^{-1}, C_2H_2:HCl:Ar = 40:44:16, W_{cat} = 0.25 g.

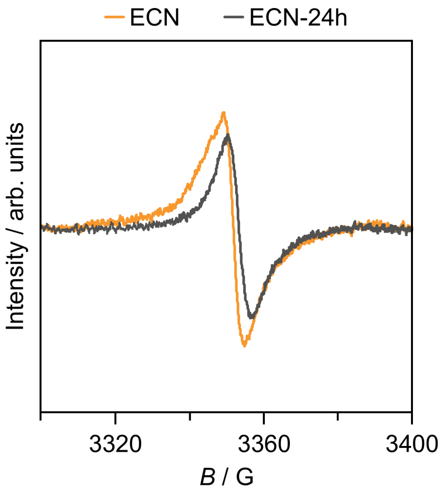

Supplementary Figure 28. CW-EPR spectra of the bare ECN support as-prepared and after use in acetylene hydrochlorination for 24 h.

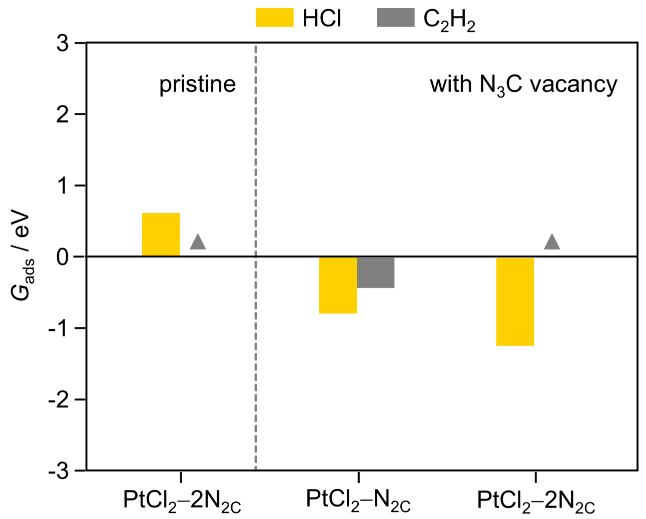

Supplementary Figure 29. Gibbs free energy, G_{ads}, of C_2H_2 and HCl on $PtCl_2$ species on pristine and defective ECN surfaces, *i.e.*, with N_3C vacancy, stabilized on one or two N_{2C} sites in the ECN support as indicated by XAS analysis (**Supplementary Table 7**). The gray triangle marks C_2H_2 physisorption on the Pt sites at a distance exceeding 3 Å as the most energetically favored configuration, indicating inhibition of C_2H_2 chemisorption.

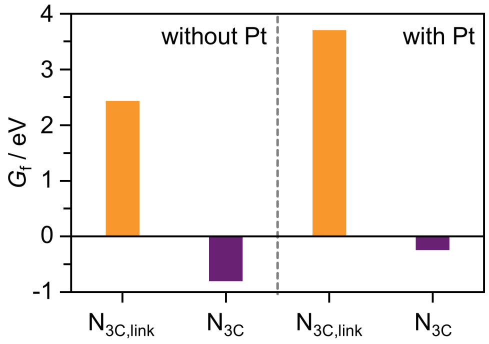

Supplementary Figure 30. Relative thermodynamic stability, obtained as relative formation Gibbs energy, G_f, of N_{3C} and $N_{3C,link}$-vacancies compared with N_{2C} ones, in the absence and presence of $PtCl_2$ single atoms.

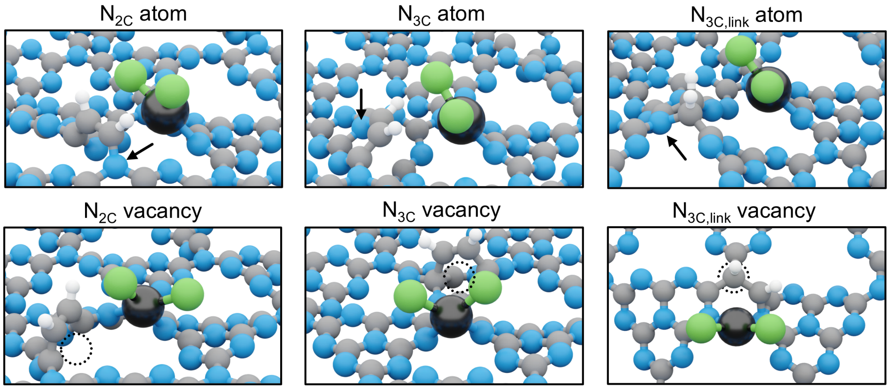

Supplementary Figure 31. Schematic representation of C_2H_2 adsorption over distinct N-atoms and N-vacancies, as respectively indicated by black arrows and dotted circles, in the ECN support for $PtCl_2$ single atoms. Color code: black Pt, blue N, green Cl, gray C, white H.

4. Supplementary references

1 Rossi, K. *et al.* Quantitative description of metal center organization and interactions in single-atom catalysts. *Adv. Mater.* **36**, e2307991, doi:10.1002/adma.202307991 (2024).

2 Chen, Z. *et al.* Stabilization of single metal atoms on graphitic carbon nitride. *Adv. Funct. Mater.* **27**, 1605785, doi:10.1002/adfm.201605785 (2017).

3 Kaiser, S. K. *et al.* Nanostructuring unlocks high performance of platinum single-atom catalysts for stable vinyl chloride production. *Nat. Catal.* **3**, 376, doi:10.1038/s41929-020-0431-3 (2020).

4 van Beek, W., Safonova, O. V., Wiker, G. & Emerich, H. SNBL, a dedicated beamline for combined in situ X-ray diffraction, X-ray absorption and Raman scattering experiments. *Ph. Transit.* **84**, 726, doi:10.1080/01411594.2010.549944 (2011).

5 Newville, M. IFEFFIT: interactive XAFS analysis and FEFF fitting. *J. Synchrotron Radiat.* **8**, 322, doi:10.1107/s0909049500016964 (2001).

6 Stoll, S. & Schweiger, A. EasySpin, a comprehensive software package for spectral simulation and analysis in EPR. *J. Magn. Reson.* **178**, 42, doi:10.1016/j.jmr.2005.08.013 (2006).

7 Giulimondi, V. *et al.* Evidence of bifunctionality of carbons and metal atoms in catalyzed acetylene hydrochlorination. *Nat. Commun.* **14**, 5557, doi:10.1038/s41467-023-41344-0 (2023).

8 Zichittella, G., Polyhach, Y., Tschaggelar, R., Jeschke, G. & Pérez-Ramírez, J. Quantification of redox sites during catalytic propane oxychlorination by operando EPR spectroscopy. *Angew. Chem. Int. Ed.* **133**, 3640, doi:10.1002/ange.202013331 (2020).

9 Tschaggelar, R. *et al.* Cryogenic 35GHz pulse ENDOR probehead accommodating large sample sizes: performance and applications. *J. Magn. Reson.* **200**, 81, doi:10.1016/j.jmr.2009.06.007 (2009).

10 Kuzin, S., Jeschke, G. & Yulikov, M. Diffusion equation for the longitudinal spectral diffusion: the case of the RIDME experiment. *Phys. Chem. Chem. Phys.* **24**, 23517, doi:10.1039/d2cp03039j (2022).

11 Kuzin, S. *et al.* Quantification of distributions of local proton concentrations in heterogeneous soft matter and non-Anfinsen biomacromolecules. *J. Phys. Chem. Lett.* **15**, 5625, doi:10.1021/acs.jpclett.4c00825 (2024).

12 Kuzin, S., Yulikov, M. & Jeschke, G. Non-commutative perturbation theory for spin dynamics explains the factorization properties of RIDME background. *J. Magn. Reson.* **365**, 107729, doi:10.1016/j.jmr.2024.107729 (2024).

13 Carberry, J. J. in *Catalysis: Science and Technology* (eds J. R. Anderson & M Boudart) Ch. 3, 131 (Springer-Verlag, 1987).

14 Mears, D. Diagnostic criteria for heat transport limitations in fixed bed reactors. *J. Catal.* **20**, 127, doi:10.1016/0021-9517(71)90073-x (1971).

15 Weisz, P. B. & Prater, C. D. Interpretation of measurements in experimental catalysis. *Adv. Catal.* **6**, 143, doi:10.1016/S0360-0564(08)60390-9 (1954).

16 Kresse, G. & Furthmüller, J. Efficient iterative schemes for ab initio total-energy calculations using a plane-wave basis set. *Phys. Rev. B* **54**, 11169, doi:10.1103/physrevb.54.11169 (1996).

17 Kresse, G. & Furthmüller, J. Efficiency of ab-initio total energy calculations for metals and semiconductors using a plane-wave basis set. *Comput. Mater. Sci.* **6**, 15, doi:10.1016/0927-0256(96)00008-0 (1996).

18 Perdew, J. P., Burke, K. & Ernzerhof, M. Generalized gradient approximation made simple. *Phys. Rev. Lett.* **77**, 3865, doi:10.1103/PhysRevLett.77.3865 (1996).

19 Grimme, S., Antony, J., Ehrlich, S. & Krieg, H. A consistent and accurate ab initio parametrization of density functional dispersion correction (DFT-D) for the 94 elements H-Pu. *J. Chem. Phys.* **132**, 154104, doi:10.1063/1.3382344 (2010).

20 Kresse, G. & Joubert, D. From ultrasoft pseudopotentials to the projector augmented-wave method. *Phys. Rev. B* **59**, 1758, doi:10.1103/PhysRevB.59.1758 (1999).

21 Blöchl, P. E. Projector augmented-wave method. *Phys. Rev. B* **50**, 17953, doi:10.1103/physrevb.50.17953 (1994).

22 Makov, G. & Payne, M. C. Periodic boundary conditions in *ab initio* calculations. *Phys. Rev. B* **51**, 4014, doi:10.1103/physrevb.51.4014 (1995).

23 Henkelman, G., Uberuaga, B. P. & Jónsson, H. A climbing image nudged elastic band method for finding saddle points and minimum energy paths. *J. Chem. Phys.* **113**, 9901, doi:10.1063/1.1329672 (2000).

24 M. J. Frisch *et al.* in *Gaussian 03, Revision C.02* (Gaussian, Inc., Wallingford CT, 2004).

25 Neese, F. The ORCA program system. *Wiley Interdiscip. Rev.: Comput. Mol. Sci.* **2**, 73, doi:10.1002/wcms.81 (2011).

26 Moulder, J. F., Stickle, W. F., Sobol, P. E. & Bomben, K. D. *Handbook of X-Ray Photoelectron Spectroscopy*. 63 (Physical Electronics, Inc., 1995).

27 McMillan, P. F. *et al.* Graphitic carbon nitride $C_6N_9H_3 \cdot HCl$: characterisation by UV and near-IR FT Raman spectroscopy. *J. Solid State Chem.* **182**, 2670, doi:10.1016/j.jssc.2009.07.030 (2009).

28 Momma, K. & Izumi, F. VESTA 3 for three-dimensional visualization of crystal, volumetric and morphology data. *J. Appl. Cryst.* **44**, 1272, doi:10.1107/s0021889811038970 (2011).
