# Supplementary material for: Tracking life and death of carbon nitride supports in platinum-catalyzed vinyl chloride synthesis
Source: Nat Commun. 2025 May 24;16:4842. doi: 10.1038/s41467-025-60169-7 (PMC12103515; doi:10.1038/s41467-025-60169-7)
Supplement: Supplementary file 2 — Description of additional supplementary file [file 41467_2025_60169_MOESM2_ESM.pdf]

Description of Additional supplementary file

Supplementary Movie 1.

Carbon nitride restructuring under reaction conditions. Upon HCl adsorption, protonation of N2C sites occurs, leading to NH_3 elimination and the subsequent formation of N_3C vacancies, which promote acetylene polymerization into coke. The video further illustrates how HCl-induced depolymerization of the carbon framework reduces C_2H_2 adsorption, contributing to catalyst deactivation.
